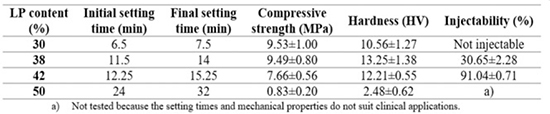# Influence of the liquid phase content and presence of hydroxypropyl methyl cellulose on the properties of a calcium phosphate bone cement

**DOI:** 10.1080/07853890.2021.1896897

**Published:** 2021-09-28

**Authors:** M. Reis, L. Figueiredo, A. Pimenta, N. Ribeiro, M. Salema-Oom, R. Colaço, A.P. Serro

**Affiliations:** a CQE, Instituto Superior Técnico, Universidade de Lisboa, Lisboa, Portugal; b DEM & IDMEC, Instituto Superior Técnico, Lisboa, Portugal; c Bioceramed, São Julião do Tojal, Portugal; d Departamento de Ortopedia, Hospital Lusíadas Lisboa, Lisboa, Portugal; e Centro de Investigação Interdisciplinar Egas Moniz (CiiEM), Egas Moniz Cooperativa de Ensino Superior, Caparica, Portugal

## Abstract

**Introduction:**

Calcium phosphate cements (CPCs) have been widely used for bone defect´s filling given their excellent biocompatibility, osteoconduction ability and ease of manipulation [1]. Nevertheless, their mechanical properties and handling performance still need to be improved to satisfy clinical requirements. Indeed, enhancing their injectability can widen its application to minimally invasive surgical procedures [2]. This work aims to investigate the effect of the liquid phase amount and presence of hydroxypropyl methyl cellulose (HPMC) on the basic properties of a commercial CPC containing a polymeric adjuvant, chitosan (Chi).

**Materials and methods:**

Starting from the original formulation containing Chi, samples with different amounts of liquid phase (LP 30%, 38%, 42%, 50%) were prepared. Additionally, for LP 38% and 42% formulations, Chi was replaced by HPMC polymer. Setting times were measured using the Vicat apparatus. After 6 days of setting, mechanical properties were studied through compression assays and Vickers hardness was measured. Injectability experiments were done and the cytotoxicity of the commercial CPC was assessed using MG63 and NIH/3T3 cell lines following the ISO 10993-5 guidelines.

**Results:**

As shown in Table 1, increasing the LP content from 30% to 50% increased the initial setting time from 6.5 min to 24 min, and the final setting time from 7.5 min to 32 min. Concerning the resistance to compression, it was lowered from 9.53 ± 1.00 MPa to 0.83 ± 0.20 MPa. Regarding materials’ hardness, it decreased by 77%. Injectability measurements showed that only 38% and 42%LP formulations could be injected. Nonetheless, while 38%LP formulation presented an injectability of 31 ± 2%, 42%LP formulation showed an injectability of 91 ± 1%. As for the addition of HPMC, for 38%LP formulation an initial setting time of 6.5 min and a final setting time of 10.5 min were measured, and for 42%LP formulation 12 min and 14.5 min, respectively. The mechanical properties presented similar values regardless the added polymer. Moreover, the formulations containing HPMC presented 69 ± 6% injectability for 38%LP and 94 ± 1% for 42%LP. Finally, when in contact with extracts of the commercial CPC for 24 h, the studied cell lines presented viability greater than 75% when compared to unexposed cells. **Discussion and conclusions:** Our study shows that LP content has a significant impact on the CPC studied properties. Effectively, for 38 and 42%LP the setting times, resistance to compression and hardness of the materials assume suitable values for their use as trabecular bone defect fillers. For the Chi CPC, 42%LP formulation is ∼200% more injectable than the 38%LP one. Even though the replacement of Chi by HPMC does not affect the mechanical properties significantly, HPMC itself seems to promote injectability, increasing it by 126%. Finally, the commercial CPC does not show a cytotoxic effect under the conditions of this assay.

**Table 1.** Effect of the LP content on the properties of the Chi containing CPC.